# A Practical Approach to Screening for *Strongyloides stercoralis*

**DOI:** 10.3390/tropicalmed6040203

**Published:** 2021-11-29

**Authors:** Luisa Carnino, Jean-Marc Schwob, Laurent Gétaz, Beatrice Nickel, Andreas Neumayr, Gilles Eperon

**Affiliations:** 1 Division of Tropical and Humanitarian Medicine, Geneva University Hospitals, Rue Gabrielle-Perret-Gentil 6, 1205 Geneva, Switzerland; jean-marc.schwob@hcuge.ch (J.-M.S.); gilles.eperon@hcuge.ch (G.E.); 2 Faculty of Medicine, University of Geneva, Rue Michel-Servet 1, 1205 Geneva, Switzerland; 3 Division of Penitentiary Medicine, Geneva University Hospitals and University of Geneva, Ch. du Petit-Bel-Air 2, 1225 Chêne-Bourg, Switzerland; Laurent.Getaz@hcuge.ch; 4 Swiss Tropical and Public Health Institute, Socinstrasse 57, 4051 Basel, Switzerland; beatrice.nickel@swisstph.ch (B.N.); andreas.neumayr@swisstph.ch (A.N.); 5 University of Basel, Klingelbergstrasse 61, 4056 Basel, Switzerland

**Keywords:** strongyloidiasis, immunosuppression, screening, corticosteroids, SARS-CoV-2

## Abstract

*Strongyloides stercoralis*, causative agent of a neglected tropical disease, is a soil-transmitted helminth which may cause lifelong persisting infection due to continuous autoinfection. In the case of immunosuppression, life-threatening hyperinfection and disseminated strongyloidiasis can develop. We propose a pragmatic screening algorithm for latent strongyloidiasis based on epidemiologic exposure and immunosuppression status that can be applied for any kind of immunosuppressive therapy. The algorithm allows the diagnosis of latent strongyloidiasis with optimal accuracy in a well-equipped setting, while for endemic settings where the complete testing array is unavailable, an empiric treatment is generally recommended. Accurate diagnosis and extensive empiric treatment will both contribute to decreasing the current neglect of strongyloidiasis.

## 1. Introduction

Strongyloidiasis is a neglected soil-transmitted intestinal helminthiasis caused by *Strongyloides stercoralis*, which is distributed worldwide in warm, humid and also semi-arid climates, infecting from 6.9% to over 30% of the population in tropical and sub-tropical countries, accounting for an estimated 614 million cases [1]. Autochthonous transmission also occurs in temperate regions such as the northern edge of the Mediterranean basin, southern parts of North America, Japan and northern Australia. Because of the parasite’s capacity to complete its life cycle within a human host, chronic asymptomatic infection can be sustained for decades, while clinical manifestations can occur long after the initial infection [2]. In the case of immunosuppression due to the use of corticosteroids, chemotherapy, biologics or HTLV-1 infection, larval reproduction can lead to strongyloides hyperinfection syndrome (SHS) and disseminated strongyloidiasis, which, untreated, have high fatality rates [3]. The majority of severe cases are reported in non-endemic countries, with only a few cases of SHS being reported in high-endemic regions. Due to the lack of diagnostic capacity and knowledge, it is likely that many severe infections remain undiagnosed in endemic countries [1]. Migration and increasing access to immunomodulatory/immunosuppressive drugs make strongyloidiasis a global clinical and public health challenge.

## 2. Diagnostic Techniques and Diagnostic Challenges

The lack of a diagnostic gold standard hampers the validation of novel diagnostic platforms and contributes to the underestimation of true disease burden. Microscopy-based direct methods (such as simple wet-smear preparation or Kato-Katz) and even conventional concentration techniques (such as the formalin-ether concentration technique (FECT)) have low sensitivity because of the irregular and often low larval output in latent strongyloidiasis. Concentration techniques specifically designed for the detection of *S. stercoralis* (such as Baermann or Koga-agar plate-culture (KAPC)) provide more reliable and more sensitive results (each method has a sensitivity in immunocompetent patients of 60%, or 77% when combined) [4]. According to some studies, nucleic acid amplification tests (such as stool PCR) are considered to have better sensitivity than the concentration techniques [5], which are less user-dependent, but expensive and rarely available in low- and middle-income countries. Serology is a more suitable screening method due to its high sensitivity (ranging from 85 to 90% depending on the manufacturing company); however, the specificity of serological tests is compromised, especially in endemic areas, by cross-reactivity with other tissue helminth infections. In addition, the sensitivity of serological tests may be reduced in immunosuppressed patients [6]. To reach optimal sensitivity, a combined approach of direct and indirect diagnostic methods is recommended [7], particularly for immunosuppressed or pre-immunosuppressed patients.

## 3. Strongyloidiasis and SARS-CoV-2 Infection: The Reason for the Increase in Awareness

Due to reported cases of coinfection with SARS-CoV-2, strongyloidiasis is currently in the limelight. Since high-dose steroid or tocilizumab treatment is recommended for severe SARS-CoV-2 presentation, SHS and disseminated strongyloidiasis have been described in patients with undetected latent strongyloidiasis [8,9]; moreover, it is possible that cases of Gram-negative bacteremia driven by *Strongyloides* hyperinfection have been overlooked by clinicians due to a lack of awareness. An increased incidence of SHS can be expected in high-endemic countries as well as more sporadic cases in low-endemic countries, where the admission to critical care for SARS-CoV-2 related ARDS is most common in south Asian, African and other ethnic minority groups [10] probably due to lower vaccination coverage, over-crowded housing conditions and untreated cardiovascular and metabolic comorbidities.

## 4. Screening Algorithm for Strongyloidiasis

Different algorithms [10,11,12,13,14] have been proposed to guide the risk assessment and management of SHS among patients with relevant epidemiological exposure, suffering from SARS-CoV-2 infection and requiring corticosteroids. Earlier algorithms assessed the risk and management of SHS in the case of immunosuppression in general [7,11] and can actually be adapted for the prescription of dexamethasone or other biological therapy in SARS-CoV-2-related complications.

Unfortunately, many of these algorithms reflect the specificities of the epidemiological context in which they were created and therefore cannot generally be applied. For example, U.S. algorithms consider the Mediterranean population to be at moderate risk for SHS and therefore eligible for empirical treatment with ivermectin, in the case of dexamethasone administration, leading to a generalized use of ivermectin if applied in Europe. Other algorithms addressing this problem include a risk assessment to carefully evaluate a possible earlier exposure to the parasite, inquiring about rural travel, poor sanitation, barefoot walking, etc. However, this represents a case-by-case management that is difficult to adapt to multiple touristic destinations or to implement in a pandemic/emergency situation. Finally, some algorithms striving for a more accurate diagnosis propose up to three stool examinations over a 10-day period in order to overcome the problem of irregular shedding of *Strongyloides* larvae, making it difficult to apply in outpatient settings (because of costs, compliance and unwillingness to bring stool samples) or in emergency situations.

**Figure 1:**
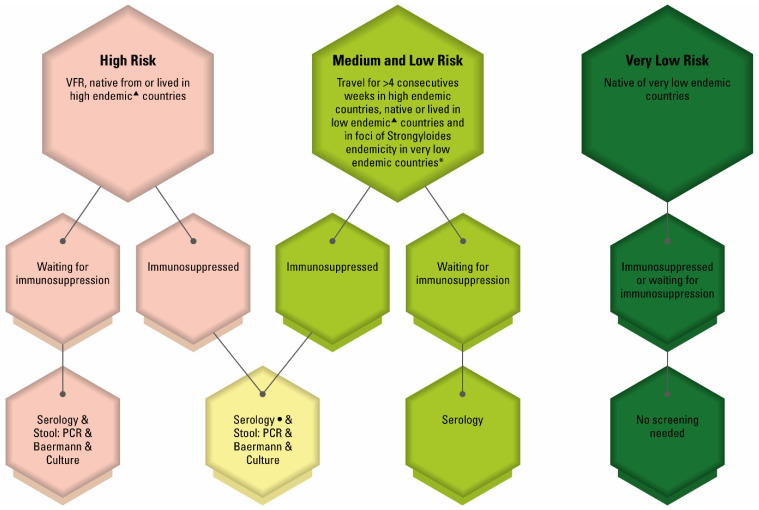
Screening algorithm for latent strongyloidiasis. Treat with ivermectin (200 μg/kg) if serology and/or stool test is positive; if PCR is not available, repeat Baermann and culture for a total of three samples; if screening tests are not available, give empiric treatment with ivermectin (200 μg/kg). VFR—visiting friends and relatives. 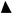 Strongyloidiasis prevalence: high-endemic countries > 5%, low-endemic countries > 0.1% and ≤5%, very low-endemic countries ≤ 0.1% (modified from Buonfrate et al., The Global Prevalence of *Strongyloides stercoralis* Infection, Pathogens 2020). * Italy, southern Spain, Australian Aboriginal communities, Southern USA and Japan. Consider a lower cutoff in the case of immunosuppression.

To overcome these shortcomings, the Department of Tropical and Humanitarian Medicine of the University Hospital of Geneva together with the Swiss Tropical and Public Health Institute (Swiss TPH) in Basel have created an algorithm (Figure 1) that can be applied during any immunosuppressive therapy, for example, chemotherapy, biological agents such as monoclonal antibodies, transplant-related immunosuppression, or HTLV-1 infection. In contrast to other published algorithms, this diagnostic flowchart is based on both the epidemiologic exposure and the immunosuppression status. In fact, while immunosuppression can reduce the sensitivity of the serology, the condition may also increase the risk of SHS where shedding of larvae is increased, facilitating the detection of the parasite with a direct diagnostic test [15]. Adapting the diagnostic process to the patient’s immune status and combining direct and indirect diagnostic tests only when needed will improve the diagnostic sensitivity for immunosuppressed patients and reduce the diagnostic costs for the non-immunosuppressed.

**Figure 2:**
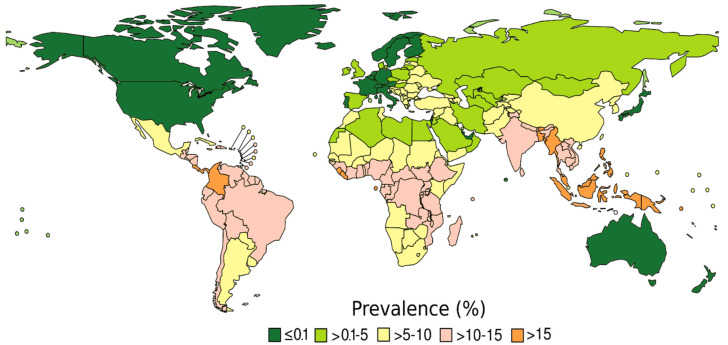
Prevalence of *Strongyloides stercoralis* infection, modified from The Global Prevalence of *Strongyloides stercoralis* Infection, Pathogens 2020.

According to a prevalence map adapted from Buonfrate et al. (Figure 2), countries are considered as high endemic with a prevalence > 5%, as low endemic if the prevalence is >0.1% and ≤5% and as very low endemic if the prevalence is ≤0.1%. Exceptions are specific foci of high endemicity in generally very low-endemic countries, such as southern Japan, northern Italy, southern USA and Aboriginal communities in Australia. Patients who have travelled for more than 4 consecutive weeks to high-endemic countries are considered to be at high risk, independent of their country of origin. The cutoff of 4 weeks is arbitrary and was based on expert opinion [16]. Serology is a useful and sensitive indirect method for the detection of strongyloidiasis in immunocompetent individuals. However, considering the lower sensitivity of serology in the case of ongoing immunosuppression [6,17], a lower serological cutoff value may be established for immunosuppressed patients. To our knowledge, only one published study has reported a different cutoff value for immunosuppressed patients [18], which cannot be generalized to the various commercial brands or to the in-house tests. An evaluation of the ROC curves of the diagnostic test used in one’s own setting should ideally be carried out with the reference laboratory to establish a cutoff value for immunosuppressed patients. However, if this cannot be done, more realistically, a lone negative serology result might be considered to be doubtful in this situation.

Repeated stool tests (up to three) are generally recommended to increase sensitivity, but this is often not performed because of logistical reasons; Baermann analysis should be performed within a short time after collection, and an interval of at least 72 h needs to be adhered to in between, making this unfeasible in the case of urgent immunosuppressive treatment [6]. If only a single stool sample is available, as is most often the case, our algorithm proposes assessment by stool PCR, KAPC and Baermann concentration in parallel, in order to increase the sensitivity of the diagnostic tests [4].

If PCR is unavailable, repeating KAPC and Baermann or other concentration techniques on two additional stool samples to reach a total of three samples will likely compensate for the loss of sensitivity caused by the absence of the PCR test. Treatment with ivermectin (200 μg/kg body weight) should be administered in the case of positive serology and/or positive stool test (from either KAPC, Baermann or PCR). Empiric treatment should be given if no diagnostic methods are available or if medical emergency does not allow waiting for results. A single dose is recommended for non-immunosuppressed patients, while 2–4 doses are necessary in the case of immunosuppression or recent exposure [19].

This algorithm is intended to be used by non-infectious disease specialists who, at the patient’s bedside, can quickly identify the patient’s risk category using the map. It is important to point out that the prevalence map is based on an estimation; if a different local prevalence is known, this should guide the diagnostic decision.

The algorithm’smain objective is to diagnose latent strongyloidiasis as accurately as possible with a combination of different diagnostic tests, particularly in immunosuppressed patients, to ensure correct management of those patients and, from an epidemiological point of view, to better understand the prevalence of this potentially fatal infection in this population in countries with low endemicity. Downsides of this algorithm include the elevated costs (due to several diagnostic tests) and the required access to all diagnostic methods, which makes it less applicable to low- and middle-income countries. In these countries, the WHO [20] favors the wide use of empiric treatment with ivermectin, to the detriment of an accurate diagnosis. This treatment is safe and well tolerated, justifying its use in the light of the risk of reactivation of latent strongyloidiasis, with its high mortality rate.

## 5. Conclusions

Due to cases of SARS-CoV-2 co-infection and the global widening use of therapeutic immunosuppression, strongyloidiasis (a neglected tropical disease) is currently in the headlines. Diagnostic algorithms for its detection need to be adapted to epidemiological and resource environments. We propose an algorithm to diagnose latent strongyloidiasis with optimal accuracy in high-resource settings, while for endemic settings where the complete testing array might be unavailable, a pragmatic approach (from repeated parasitological examinations to an empiric treatment) is recommended. Accurate diagnosis and extensive empiric treatment will both contribute to decreasing the current neglect of strongyloidiasis.

## Data Availability

Not applicable.
